# Adverse health manifestations in the hands of vibration exposed carpenters - a cross sectional study

**DOI:** 10.1186/s12995-021-00305-3

**Published:** 2021-04-29

**Authors:** Eva Tekavec, Lotta Löfqvist, Anna Larsson, Karin Fisk, Jakob Riddar, Tohr Nilsson, Catarina Nordander

**Affiliations:** 1 Division of Occupational and Environmental Medicine, Lund University, Lund, Sweden; 2 Division of Sustainable Health and Medicine, Department of Public Health and Clinical Medicine, Umeå University, Umeå, Sweden

**Keywords:** Painters, White fingers, Neurosensory affection, Carpal tunnel syndrome, Hand arm vibration syndrome, Vibration exposure

## Abstract

**Background:**

Despite EU regulatory standards, many workers suffer injury as a result of working with hand-held vibrating tools. Our aim of this study was to confirm whether carpenters, a highly exposed group, suffer more injuries to their hands than painters, a group assumed to be less exposed to vibration.

**Methods:**

193 carpenters (participation rate 100%) and 72 painters (participation rate 67%), all men, answered a questionnaire and underwent a clinical examination to identify manifestations of neural and vascular origin in the hands. *Neurosensory affection* was defined as having at least one symptom in the fingers/hands (impaired perception of touch, warmth, or cold, impaired dexterity, increased sensation of cold, numbness or tingling, or pain in the fingers/hands when cold) and at least one clinical finding (impaired perception of touch, warmth, cold, vibration, or two-point discrimination). Logistic regression was used to calculate odds ratios (OR) and 95% confidence intervals (CI).

**Results:**

*Neurosensory affection* was fulfilled for 31% of the carpenters and 17% of the painters*,* age-adjusted OR 3.3 (CI 1.6–7.0). Among carpenters with *neurosensory affection* 18% reported interference with daily life activities, the most common symptoms being increased sensation of cold, numbness and pain in the fingers/hands when cold, the most common clinical findings were impaired perception of touch and vibration. *Neurosensory affection* was found in 12% of young carpenters (≤ 30 years old)*.* No difference was found in the prevalence of white fingers between carpenters and painters.

**Conclusions:**

Carpenters showed more symptoms and clinical findings of *neurosensory affection* than painters, probably due to vibration exposure. Also young carpenters showed signs of *neurosensory affection,* which indicates that under current conditions workers at these companies are not protected against injury. This underlines the importance of reducing exposure to vibration and conducting regular medical check-ups to detect early signs of neural and vascular manifestations indicating hand-arm vibration injuries. Special attention should be given to symptoms of increased sensation of cold, pain in the fingers when cold, and numbness, as these were the most common initiating ones, and should be addressed as early as possible in the preventive sentinel process. It is also important to test clinically for small- and large-fibre neuropathy, as the individual may be unaware of any pathology.

## Background

Despite EU regulations [1] many workers handling vibrating tools get injured. Vibration injury is in fact, the most common diagnosis among Swedish men receiving compensation for occupational injuries [2]. Approximately 400,000 workers, corresponding to 13% of all men and 3% of all women in the Swedish workforce, are exposed to hand-held vibrating tools for at least two hours a day. In the construction sector, as many as 70% report daily exposure to vibrating tools [3].

Vibration injury is characterised by adverse health manifestations in digital nerves and vessels. Neural symptoms include impaired perception of touch and temperature and numbness (negative manifestations), additive neurosensory sensations (positive manifestations); tingling and increased sensation of cold, and provocable manifestations; pain in the finger/hands when cold or when the nerves are compressed. Impaired dexterity, impaired grip strength, sweating and shaking of the hands are often co-reported [4–6]. Manual handling of vibrating tools is ergonomically demanding and vibration exposure in combination with forceful grips in non-neutral wrist positions makes the median nerve prone to entrapment in the carpal tunnel [7]. Pain in the upper extremity is often co-reported with symptoms from the hands [8, 9]. Taken together these symptoms are often referred to as hand-arm vibration syndrome (HAVS) with neural, vascular and musculoskeletal components [10, 11]. The condition leads to considerable negative health impact, and little or no improvement is expected even if vibration exposure ceases [11]. Many patients report effects on their daily life activities due to impaired upper extremity function [12–14].

Carpenters constitute a large group in the construction sector, and the frequency of HAVS in this group has been shown to be twice that in workers not exposed to vibration [15]. Carpenters use vibrating tools such as impact drills, impact power wrenches and grinders. Endeavors to decrease the ergonomic load have resulted in the development of lighter tools, but these tools may emit more vibration than the heavier tools.Furthermore, the number of workers in the construction sector using vibrating tools is increasing [2]. Although statistics are available concerning the number of workers claiming compensation for occupational injuries, it is not known how prevalent neurosensory or vascular manifestations are among carpenters. Few studies have been carried out to determine whether exposure to vibration is still hazardous, or whether working conditions have improved. This information could be useful in making screening more efficient, and allowing measures to prevent further injury from vibration to be instigated.

The aim of the study was to assess the occurrence of adverse health manifestations in hands of neural and vascular origin among carpenters, who are heavily exposed to vibrating tools and to compare it with the occurrence among painters with the same ergonomic conditions, but presumably with much less vibration exposure. Furthermore, we investigated which symptoms appeared first, and which were the most prevalent, and the extent to which symptoms and clinical findings occur in combination. In order to determine whether improvements have been made in working conditions, we also investigated manifestations of injury in the younger carpenters in the group.

## Subjects and methods

### Study design

In this cross sectional study, we examined carpenters at their work sites, and painters at their worksites or at the Occupational and Environmental Medicine Clinic. Carpenters working for two medium-sized and two large construction companies were examined at 18 different work sites between April 2016 and April 2018. All the carpenters at these work sites were invited to take part in the study. The painters were recruited from three medium-sized painting companies, and were invited to participate at staff meetings, as they worked at different, smaller work sites. They were examined between May 2018 and September 2019. The participants were instructed not to use nicotine or vibrating tools one hour prior to the clinical examination. Their finger temperature was measured on arrival, and if it was below 28 °C the hands were heated with warm pads until start of the clinical examination. They then filled in a questionnaire on a laptop (30–45 min). The participants were able to go back and change an answer, but not to skip questions. Some answers generated follow-up questions. An examiner was present in the room to answer any queries. All participants then underwent a clinical examination.

To assess the generalisability of the results, a shortened questionnaire was sent by post to all the carpenters who worked at the same construction companies, but at other work sites, for three of the four companies included in this study. This questionnaire was also sent to male carpenters at other construction companies, randomly selected from a list provided from the relevant trades union. Two reminders were sent.

### Study population

**Fig. 1:**
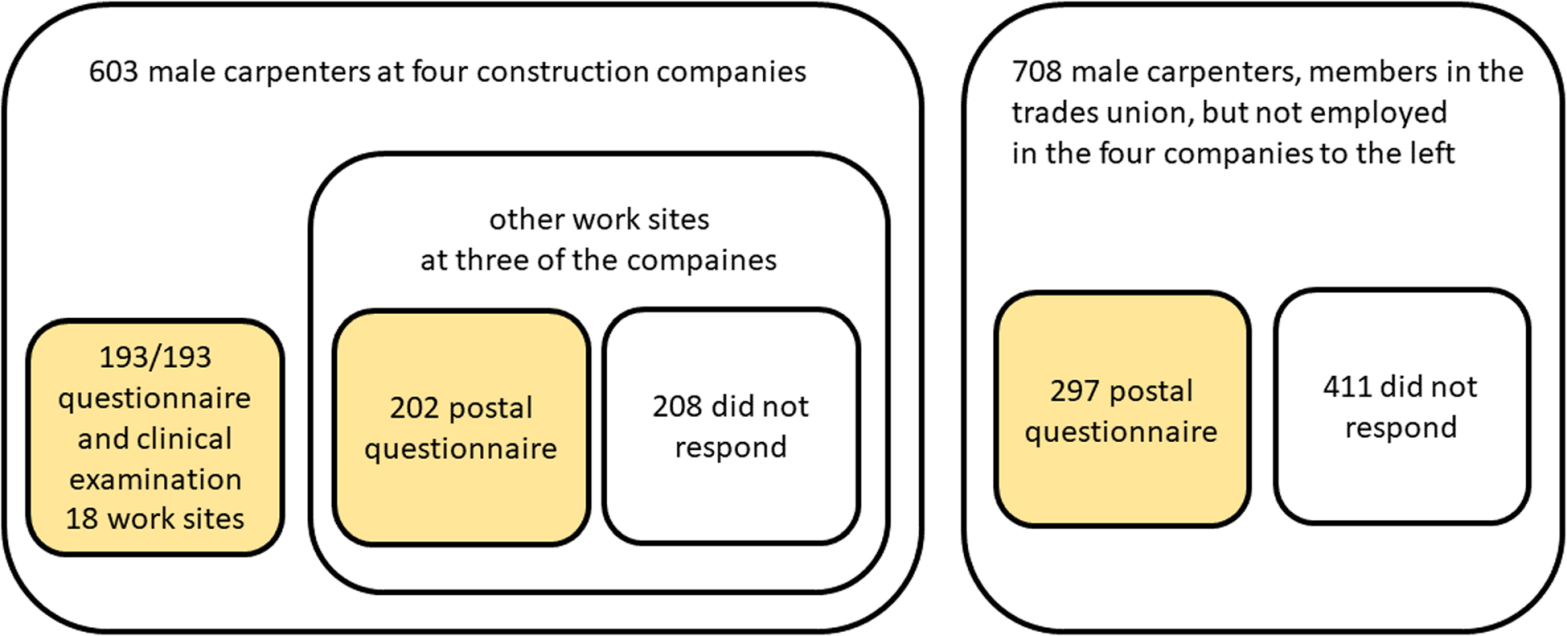
The different groups of male carpenters

The study included all 193 invited male carpenters (response rate 100%; one female carpenter was examined but was excluded from the data analyses). There were 139 male painters at the companies included in the study, 108 of whom attended the staff meetings, and were invited to participate in the study. The others could not be contacted. Of these 108, 72 agreed to participate in the study (participation rate 67%). All eight female painters invited to take part in the study participated but were not included in the data analyses. The shortened postal questionnaire was returned by 202 of the 410 male carpenters at the construction companies chosen for the study (response rate 49%), and by 297 of 708 at other companies (response rate 42%).The different groups of male carpenters are visualised in Fig. 1.

### Job tasks and vibration exposure

Carpenters, in the International Standard Classification of Occupations (ISCO) 7124, perform different work tasks in the construction or repair of buildings. According to the results of the questionnaire, common work tasks with vibration exposure among carpenters were framework, formwork and demolition. Plastering was the most common work task, but with lower vibration exposure. Carpenters used numerous vibrating tools both with low and high frequencies and impact tools. The most common ones were screw drivers, impact drills and impact power wrenches, with interquartile ranges of vibration levels of 3–4, 8–15 and 5–9 m/s^2^ respectively as measured according to the ISO 5349 standard. Painters, ISCO 7141, usually did not use vibrating tools, however e.g. long reach sanders (vibration level interquartile range 3–4 m/s^2^) could cause some exposure to vibration. Preliminary data concerning the vibration exposure A(8) indicates that the Daily Action Value (2.5 m/s^2^) regularly was exceeded by carpenters but only occasionally by painters. The Daily Exposure Limit Value (5 m/s^2^) appeared to only be exceeded by carpenters.

### Outcome assessments

#### Symptom questionnaire

The following questions were asked regarding neurosensory symptoms in the fingers/hands: “Do you experience: sensation of cold; impaired perception of touch; impaired perception of warmth; impaired perception of cold; numbness or tingling when not working with vibrating tools; pain when cold; a tendency to drop things; or difficulty buttoning clothes?” The possible responses were: “Not at all”, “Insignificant”, “Somewhat” or “Quite a lot”. The alternatives “Somewhat” or “Quite a lot” were considered a positive response. If a positive response was given to any of these questions, the participant was asked which of these symptoms that appeared first and when. More than one symptom could be given.

To assess symptoms of Raynaud’s phenomenon*,* we asked: “Do one or more of your fingers turn white when you are exposed to cold or dampness?” A colour photograph was shown to facilitate recognition of the condition. Possible responses were “No” or “Yes”. Those who responded “Yes” were considered to have white fingers, and were asked to draw the extent of their worst occurrence on a diagram of a hand, and to report the frequency of this symptom: “Once a month or less”, “Once a week”, “Once a day”, “Several times a day”, “All the time”. They were also asked when their symptoms of white fingers started.

Participants who reported neurosensory symptoms or *white fingers* were asked whether any of these conditions influenced the activities of their daily life, at work or during leisure time. Additional questions were asked concerning symptoms in the hands: reduced strength, shaking, sweating cramps, pain in the fingers/hands/forearms/elbows, and pain in the neck/shoulders, with possible responses: “Not at all”, “Insignificant”, “Somewhat” or “Quite a lot”. The alternatives “Somewhat” or “Quite a lot” were considered a positive response. The shortened questionnaire included the same questions as the main questionnaire concerning neurosensory symptoms, white fingers and other symptoms in the upper limb.

#### Clinical examination

After completing the questionnaire, the participants underwent testing of finger perception of dig II and V bilaterally [16–20]. This was performed in a quiet room at room temperature. We recorded whether the participant could detect the stimulus. The participant was asked to place both hands on a table, with the palms facing upwards, and to close his eyes. Perception of touch was tested with a Semmes Weinstein Monofilament® (detection threshold 3.61, calibrated such that 0.271 g of force is required to bend it when touched on the skin of the finger) [21]. The participant was asked to report when he felt the touch of the filament when it was applied to the finger pulps, once on each finger. For two-point discrimination (2PD) we performed a static 2PD test with a two-point discriminator (separation 5 mm); seven correct responses out of ten attempts was considered normal. The ability to distinguish between cold and warmth was tested with temperature rolls at 25 °C and 40 °C (RollTemp® instrument), randomly applied to the middle phalanges [22, 23]. Perception of vibration was tested with a VibroSense®* equipment. The participants were given information on the test procedure and asked to wear earmuffs as high amplitudes at high frequencies generate a noise. Vibration perception thresholds (VPT) were recorded at 125 Hz and 250 Hz. Vibration thresholds exceeding one standard deviation (SD) above the mean value in the age-adjusted reference material were defined as impaired perception of vibration according to standards of the method [24]. When impaired perception of vibration was found in the digits, the lower extremity was examined, by applying vibrations of 128 Hz with a Vibratip® device at the base of the proximal phalange of the big toe.

Specific nerve entrapment tests were also performed to diagnose nerve entrapment in the neck or upper extremities, including tests of neck mobility, the strength of selected muscles and provocation of the brachial plexus, as well as the median, radial and ulnar nerves.

### Individual characteristics

The questionnaire also included questions on age, sex, nicotine use, medication, concurrent diseases, hearing impairment and doctor’s diagnosis of carpal tunnel syndrome. Questions were also included to obtain information on previous injuries to the neck, arm or hand that required hospital care. The responses to this question were reviewed by one of the authors (ET), and if these injuries could have caused neurosensory impairment in the hands, they were noted as “injury relevant to hands”. Questions were also included on previous and present work tasks, employment, vibration tools used, and the participants’ views on the company’s safety policy were also included. These results will be presented elsewhere.

### Data processing and statistics

Symptoms reported in the questionnaire were combined with clinical findings to indicate neural manifestation. These were staged by applying the International Consensus Criteria (ICC) [25], with some modifications. Stage N1: numbness and/or tingling of fingers; stage N2: as in stage 1, and with loss in two of three sensory modalities; perception of touch, temperature and vibration; and stage N3: as in stage 2, and with symptoms of impaired dexterity (a tendency to drop things or difficulty buttoning clothes) and reduced 2PD. For comparison, we also graded symptoms and findings according to the Stockholm Workshop Scale (SWS) [26], with some modifications. Stage 1SN: implying numbness and/or tingling; stage 2SN: as in stage 1SN and reduced perception of touch; and stage 3SN: as in stage 1SN and impaired 2PD. In addition, we defined the clinical condition *neurosensory affection* (a de novo definition)*,* as the combination of at least one symptom in the fingers/hands (impaired perception of touch, warmth or cold, impaired dexterity, increased sensation of coldness, numbness or tingling, or pain when cold) and at least one clinical finding: impaired perception of touch, warmth, cold, or vibration, or impaired 2PD.

Vascular manifestation of HAVS was evaluated by calculating Griffin scores for participants who had reported white fingers and indicated this on a hand diagram [27]. The right and left hands were graded separately, and the result for the hand with the highest score (i.e. the highest number of blanching phalanges) was used. The severity of finger blanching was then graded according to the vascular component of ICC [25]. Stage V1: Griffin score 1–4; stage V2: Griffin score 5–12; and stage V3: Griffin score > 12 [25]. We also graded symptoms and findings according to SWS, as above. Stage 1 V: occasional attacks affecting only distal phalanges; stage 2 V: occasional attacks affecting distal/middle phalanges on one or more fingers; stage 3 V: frequent attacks affecting all phalanges on most fingers.

Logistic regression, with calculations of odds ratios and 95% confidence intervals, was used to compare the point prevalences of the different outcomes in carpenters and painters. These were reported as crude and age-adjusted values. Finally, participants with conditions that could affect the outcomes (diabetes, cardiovascular, thyroid or rheumatic disease, impaired perception of vibration in the foot, or peripheral nerve entrapment in upper extremity/neck (except for carpal tunnel syndrome) were excluded to test whether the results were influenced by such conditions (a sensitivity analysis). Two participants (both painters) reported the debute of white fingers before the age of 18 (i.e. before working age) and were excluded from all analyses concerning white fingers.

Among carpenters who were found to have *neurosensory affection* in the hands, the percentage of carpenters that indicated a specific symptom as the first occurring one, as well as the prevalence of influence on daily life activities, were calculated. Further, to investigate possible correlations between symptoms and clinical findings, the prevalence of different pairwise combinations of symptoms among these carpenters were calculated. To determine whether current exposure is associated with the same risks as previously, we also calculated the prevalence of symptoms, findings and clinical conditions in carpenters aged 30 years and younger.

Based on the responses to the shortened postal questionnaire we calculated the prevalence of adverse neurosensory and vascular health manifestations in the fingers/hands of carpenters at the same companies, that were not examined, as well as carpenters at other companies, to assess generalisability of our results.

SPSS Statistics for Windows, Version 24.0 (IBM Corp., released 2016, Armonk, NY, USA) was used for all statistical calculations.

Possible nerve entrapment was defined according to criteria modified from Nordander et al. 2009 and Ohlsson et al. 1994 [28, 29], see Table 1.

**Table 1:** The case criteria for nerve entrapments according to criteria modified from Nordander et al. 2009 and Ohlsson et al. 1994

Nerve entrapment	Criteria
Radiating neck complaints	Pain radiating from the neck to the upper extremity, and limited/restricted neck movement, and radiating pain provoked by neck movement, and muscle weakness of the upper limb.
Thoracic outlet syndrome	Pain radiating to the upper extremity, in the distribution of the ulnar nerve, and paraesthesia in the distribution of the ulnar nerve, and positive Roos test (increase in subjective symptoms, not only fatigue), and intense tenderness over the brachial plexus; diagnosis only if cervical syndrome is not present.
Pronator teres syndrome	Pain in the medial/proximal part of the forearm, and local tenderness over the edge of the pronator teres, and decreased strength in pronation or flexion of the wrist or the distal phalanges of digit I-II.
Radial tunnel syndrome	Pain in the elbow during rest, and tenderness about 2–3 in. distally of the lateral epicondyle, and pain in the proximal, lateral part of the forearm, and pain on resisted supination.
Ulnar nerve entrapment at the elbow	Pain and paraesthesia or numbness in the distribution of the ulnar nerve, and positive Tinel’s sign over the cubital tunnel.
Ulnar nerve entrapment in the wrist	Pain and paraesthesia or numbness in the distribution of the ulnar nerve, and positive Tinel’s sign over Guyon’s tunnel (volar/ulnar at the wrist) or decreased strength in spreading the fingers.
*Carpal tunnel syndrome*	Previous surgery for carpal tunnel syndrome, or numbness or tingling in digit I-III and a positive nerve entrapment test (Phalen’s test or Tinel’s sign).

## Results

The individual characteristics of the participants are presented in Table 2. The average age of the painters were somewhat higher than the carpenters. Injury relevant to hands was more common among the carpenters.

**Table 2:** Individual characteristics of the carpenters and painters (all male)

	Carpenters *N* = 193	Painters *N* = 72
Age, mean (range), (y)	40 (17**–**65)	46 (23**–**68)
Seniority in the occupation mean (range) (y)^a^	20 (0**–**50)	24 (3**–**54)
Tobacco use, N (%)	86 (45)	32 (44)
Diabetes, N (%)	5 (3)	1 (1)
Cardiovascular disease, N (%)	15 (8)	8 (11)
Hypothyreosis, N (%)	2 (1)	1 (1)
Rheumatic disease, N (%)	3 (2)	2 (3)
Injury relevant to hands, N (%)	47 (24)	6 (8)
Hearing impairment, N (%)	56 (29)	9 (13)

^a^Data missing from 21 carpenters and 4 painters

### Adverse health manifestations in hands – comparison of carpenters and painters

#### Symptoms

Carpenters reported a higher occurrence of impaired perception of touch, impaired perception of warmth, increased sensation of cold, and pain in fingers when cold, than painters (Table 3). After excluding individuals with conditions that could affect the outcome, the ORs remained high, but only increased sensation of coldness remained statistically significant. Reports of white fingers did not differ significantly between the carpenters and painters. Pain in the neck/shoulders was less common among carpenters than painters, but after age adjustment this difference was not statistically significant.

**Table 3:** Prevalence of adverse health manifestations among carpenters (*N =* 193) and painters (*N =* 72). Odds ratios (OR) and 95% confidence intervals (CI) calculated by logistic regression (Statistically significant differences are shown in bold face)

	Carpenters N (%)	Painters N (%)	Crude OR (CI)	Age-adjusted OR (CI)	Sensitivity analysis^b^ OR (CI)
**Symptoms**					
***Negative neural manifestations***					
Impaired perception of touch	28 (15)	3 (4)	**3.9 (1.1–13.3)**	**4.4 (1.3–15.0)**	4.2 (0.9–19.0)
Impaired perception of cold	19 (10)	3 (4)	2.5 (0.7–8.8)	2.7 (0.8–9.6)	2.6 (0.5–12.1)
Impaired perception of warmth	23 (12)	2 (3)	**4.7 (1.1–20.6)**	**5.7 (1.3–25.4)**	4.1 (0.9–18.8)
Tendency to drop things	23 (12)	3 (4)	3.1 (0.9–10.7)	3.3 (0.9–11.5)	2.9 (0.6–13.3)
Difficulty buttoning clothes	13 (7)	2 (3)	2.5 (0.6–11.5)	3.0 (0.7–14.0)	1.7 (0.3–8.6)
Impaired grip strength	35 (18)	9 (13)	1.5 (0.7–3.4)	1.7 (0.8–3.9)	1.1 (0.4–2.7)
***Positive neural manifestations***					
Increased sensation of cold	67 (35)	14 (19)	**2.2 (1.1–4.2)**	**2.5 (1.3–5.0)**	**2.2 (1.1–4.7)**
Numbness or tingling	51 (26)	16 (22)	1.3 (0.7–2.4)	1.4 (0.7–2.6)	1.3 (0.6–2.8)
Cramp	26 (14)	8 (11)	1.2 (0.5–2.9)	1.2 (0.5–2.9)	0.8 (0.3–2.1)
Shaking	22 (11)	4 (6)	2.2 (0.7–6.5)	2.4 (0.8–7.2)	2.2 (0.6–8.2)
Hand sweat	35 (18)	8 (11)	1.7 (0.8–4.0)	1.5 (0.7–3.6)	1.5 (0.6–3.8)
***Provoked manifestations***					
White fingers at cold or dampness ^a^	18 (9)	6 (8)	1.1 (0.4–2.9)	1.7 (0.6–4.6)	2.2 (0.6–7.8)
Pain in fingers when cold	47 (24)	6 (8)	**3.5 (1.4–8.7)**	**3.6 (1.4–8.8)**	2.5 (1.0–6.5)
***Musculoskeletal symptoms***					
Hand/elbow pain	60 (31)	21 (29)	1.1 (0.6–1.9)	1.1 (0.6–2.1)	1.0 (0.5–2.0)
Neck/shoulder pain	67 (35)	36 (50)	**0.5 (0.3–0.9)**	0.6 (0.3–1.0)	0.6 (0.3–1.1)
**Clinical findings**					
Impaired perception of touch	60 (31)	25 (35)	0.9 (0.5–1.5)	1.3 (0.7–2.6)	1.6 (0.7–3.3)
Impaired perception of cold	12 (6)	6 (8)	0.7 (0.3–2.0)	0.9 (0.3–2.5)	0.5 (0.2–1.6)
Impaired perception of warmth	7 (4)	3 (4)	0.9 (0.2–3.4)	0.9 (0.2–3.6)	1.0 (0.2–5.4)
Impaired 2PD	17 (9)	2 (3)	3.4 (0.8–15.1)	4.1 (0.9–18.4)	2.5 (0.5–12.0)
Increased VPT at 125 Hz	19 (10)	6 (8)	1.2 (0.5–3.1)	1.6 (0.6–4.6)	1.7 (0.5–5.4)
Increased VPT at 250 Hz	38 (20)	8 (11)	2.0 (0.9–4.4)	2.0 (0.9–4.6)	2.0 (0.8–5.2)
Increased VPT at 125 and 250 Hz*	42 (22)	10 (14)	1.7 (0.8–.3.7)	2.0 (0.9–4.5.)	2.1 (0.9–5.2)

^a^Two painters excluded due to debut of white fingers before age 18

^b^Excluding participants with diabetes, cardiovascular, thyroid or rheumatic disease, impaired perception of vibration in the foot, or peripheral nerve entrapment in upper extremity/neck (except for carpal tunnel syndrome)

#### Clinical findings

Carpenters exhibited twice the point prevalence of increased vibration perception thresholds at 250 Hz (Table 3), and three times the point prevalence of impaired 2PD, compared to painters, neither of which were statistically significant.

#### Neural and vascular manifestations

No statically significant differences were found between carpenters and painters regarding manifestations when comparing the prevalence of ICC stage 2 or more, or SWS stage 2SN or more (Table 4). Instead, when combining symptoms and findings according to our definition of *neurosensory affection,* we found an age-adjusted OR of 3.3 (CI 1.6–7.0). The OR remained increased excluding individuals with conditions that could affect the outcome. Concerning vascular manifestations, the ORs were high but there were few cases and the CIs were broad. The prevalence of carpal tunnel syndrome was about 10% in both groups (Table 4). Other forms of nerve entrapment were rare.

**Table 4:** Clinical conditions among 193 carpenters and 72 painters. Odds ratios (OR) and 95% confidence intervals (CI) calculated by logistic regression (Statistically significant differences are shown in bold face)

	Carpenters N (%)	Painters N (%)	Crude OR (CI)	Age-adjusted OR (CI)	Sensitivity analysis^b^ OR (CI)
**Neural manifestations**					
ICC ≥ N2	11 (6)	2 (3)	2.1 (0.5**–**9.8)	2.8 (0.6**–**13.2)	n.a.
SWS ≥ 2SN	32 (17)	7 (10)	1.8 (0.8**–**4.4)	2.3 (0.9**–**5.6)	**3.2 (1.0–10.1)**
*Neurosensory affection*	60 (31)	12 (17)	**2.3 (1.1–4.5)**	**3.3 (1.6–7.0)**	**3.5 (1.5–8.3)**
Nerve entrapment (all except carpal tunnel syndrome)	25 (13)	8 (11)	1.2 (0.5**–**2.8)	1.3 (0.6**–**3.2)	n.a.
Carpal tunnel syndrome	20 (10)	8 (11)	0.9 (0.4**–**2.2)	1.1 (0.4**–**2.6)	1.6 (0.5**–**5.2)
**Vascular manifestations** ^**a**^					
ICC ≥ V2	9 (5)	1 (1)	3.4 (0.4**–**27.1)	5.6 (0.7**–**47.2)	6.1 (0.7**–**55.8)
SWS ≥ V2	10 (5)	2 (3)	1.9 (0.4**–**8.7)	2.8 (0.6–13.5)	6.0 (0.7**–**52.5)

^a^Data missing from two carpenters due lack of hand diagram, and data excluded from two painters due to debut of *white fingers* before age 18

^b^Excluding participants with diabetes, cardiovascular, thyroid or rheumatic disease, impaired perception of vibration in the foot, or peripheral nerve entrapment in upper extremity/neck (except for carpal tunnel syndrome)

*n.a*. not applicable

### The clinical picture

Among carpenters with *neurosensory affection* or white fingers, 74% fulfilled the criteria for *neurosensory affection*: 13% reported white fingers only, and 13% had a combination. Two carpenters reported white fingers as the earliest symptom, while all other carpenters reported that symptoms of impaired neurosensory function occurred first. Among the 60 carpenters who fulfilled our criteria for *neurosensory affection,* the most common symptoms were increased sensation of coldness (67%), numbness or tingling (55%) and pain when cold (45%) (Table 5). These symptoms were also the most common early symptoms (Table 6).

**Table 5:** *Existing co-occurring* neural and vascular *manifestations* among the 60 carpenters with *neurosensory affection*. Bold face denotes the prevalence of each symptom and finding

	IPT	IPC	IPW	TDT	DBC	IGS	ISC	N	C	S	HS	WF	PWC	MF	TC	TW	2PD	125	250	VIB
	%	%	%	%	%	%	%	%	%	%	%	%	%	%	%	%	%	%	%	%
**Symptoms**																				
Impaired perception of touch (IPT)	**30**																			
Impaired perception of cold (IPC)	15	**20**																		
Impaired perception of warmth (IPW)	24	8	**32**																	
Tendency to drop things (TDT)	13	7	10	**22**																
Difficulty buttoning clothes (DBC)	7	2	4	10	**12**															
Impaired grip strength (IGS)	13	4	11	15	7	**30**														
Increased sensation of cold (ISC)	24	17	26	17	9	17	**67**													
Numbness (N)	15	9	20	13	4	17	35	**55**												
Cramp (C)	11	4	11	10	2	7	11	15	**20**											
Shaking (S)	7	2	7	9	4	9	11	13	9	**17**										
Hand sweat (HS)	11	7	13	12	4	7	20	15	7	9	**25**									
White fingers (WF)	7	4	9	3	2	4	13	13	4	4	2	**15**								
Pain when cold (PWC)	15	13	15	13	4	9	15	26	9	7	9	9	**45**							
**Clinical findings**																				
Monofilament! (MF)	20	13	22	17	7	15	52	41	15	15	15	15	35	**72**						
*RollTemp* Cold (TC)	11	7	9	10	2	4	11	7	7	0	2	2	7	9	**17**					
*RollTemp* Warm (TW)	7	7	7	2	0	2	9	4	2	0	2	0	7	7	4	**8**				
Two-point discrimination (2PD)	11	4	9	7	4	11	15	13	4	4	4	7	11	15	2	4	**25**			
Vibration at 125 Hz (125)	7	7	9	3	2	30	15	13	2	2	7	7	11	9	0	2	7	**17**		
Vibration at 250 Hz (250)	17	13	17	7	2	11	30	26	9	7	13	9	17	17	4	4	7	13	**37**	
Vibration at 125 or 250 Hz (VIB)	17	13	20	7	2	11	33	30	9	7	15	11	20	20	4	4	9	17	37	**40**

**Table 6:** Earliest symptom among the 60 carpenters with *neurosensory affection*

Symptom	N (%)
Increased sensation of cold	28 (47)
Numbness	24 (40)
Pain in fingers/hands when cold	14 (23)
Impaired perception of warmth	7 (12)
Impaired perception of touch	5 (8)
Impaired perception of cold	4 (7)
Impaired dexterity - tendency to drop things	4 (7)
Impaired dexterity - difficulty buttoning clothes	4 (7)

The most common clinical findings among the carpenters with *neurosensory affection* were impaired perception of touch (72%), and increased VPT (40%), where an impairment at 250 Hz was more common than at 125 Hz (37 and 17%, respectively; Table 5). The most common pairwise combinations of symptoms were: increased sensation of coldness with numbness or tingling (35%), and increased sensation of coldness with impaired perception of warmth (26%). The most common pairwise combinations of findings with symptoms were: impaired perception of touch with increased sensation of coldness (52%), numbness or tingling (41%), and pain when cold (35%). Impaired perception of touch and increased VPT (20%) was the most frequent combination of clinical findings. Combinations of white fingers with other symptoms or findings were rare. The majority of carpenters with *neurosensory affection* (54%), had these symptoms daily, and almost one fifth (18%) reported discomfort to such an extent that it influenced their daily life activities: work 13%, leisure time 15% (not in table).

Among the 18 carpenters that had white fingers, 2% reported having symptoms on a daily basis, 3% that these symptoms affected their work and 1% reported affection on leisure activities, and.

### Young carpenters

Among the 60 carpenters aged 30 years or less, 28 (47%) reported at least one neurosensory symptom indicating HAVS, most commonly increased sensation of coldness (28%) or pain in the hands when cold (25%; Table 7). One fifth had increased VPT and 12% fulfilled our criteria for *neurosensory affection.* In addition, one third reported neck/shoulder pain.

**Table 7:** Prevalence of adverse health manifestations among 60 young carpenters (≤ 30 years old)

	N (%)
**Symptoms**	
*Negative neural manifestations*	
Impaired perception of touch	5 (8)
Impaired perception of cold	3 (5)
Impaired perception of warmth	3 (5)
Tendency to drop things	6 (10)
Difficulty buttoning clothes	2 (3)
*Positive neural manifestations*	
Increased sensation of cold	17 (28)
Numbness or tingling	10 (17)
*Provoked manifestations*	
White fingers at cold or dampness	1 (2)
Pain in hands when cold	15 (25)
*Musculoskeletal findings*	
Hand/elbow pain	16 (27)
Neck/shoulder pain	18 (30)
**Clinical findings**	
Impaired perception of touch	5 (8)
Impaired perception of cold	3 (5)
Impaired perception of warmth	2 (3)
Increased 2PD	3 (5)
Increased VPT at 125 Hz	5 (8)
Increased VPT at 250 Hz	11 (18)
Increased VPT at 125 & 250 Hz	12 (20)
**Clinical conditions**	
Carpal tunnel syndrome	2 (3)
*Neurosensory affection*	7 (12)

### Adverse health manifestations in carpenters’ hands in general

The carpenters that were not examined, but who answered the shortened questionnaire, reported a higher prevalence of almost all symptoms than the carpenters examined in this study (Table 8). No major differences were seen in symptoms between the two non-examined groups of carpenters (at the same companies as those examined and at other companies).

**Table 8:** Prevalence (%) of symptoms among the examined carpenters (*N =* 193), non-examined carpenters at the same companies, but working at other sites from the ones that were examined (*N =* 202), and at other companies (*N* = 297) (all men)

	Carpenters - examined	Carpenters - not examined - same companies	Carpenters - not examined - other companies
	N (%)	N (%)	N (%)
Age, mean (min.-max.)	40 (17–65)	46 (23–67) ^a^	45 (19–66) ^b^
*Negative neural manifestations*			
Impaired perception of touch	15	25	25
Impaired perception of cold	10	17	17
Impaired perception of warmth	12	23	18
Tendency to drop things	12	18	23
Difficulty buttoning clothes	7	14	19
Impaired grip strength	18	28	33
*Positive neural manifestations*			
Increased sensation of cold	35	49	45
Numbness or tingling	26	46	41
Cramp	14	33	28
Shaking	14	21	23
Hand sweat	18	18	21
*Provoked manifestations*			
White fingers when cold or damp	9	32^c^	30^d^
Pain when cold	24	44	46
*Musculoskeletal manifestations*			
Hand/elbow pain	31	50	53
Neck/shoulder pain	35	61	57

^a^Data missing from 14 subjects

^b^Data missing from 5 subjects

^c^Data missing from 68 subjects

^d^Data missing from 52 subjects

## Discussion

### Main findings

One third of all the carpenters examined in this study had neural manifestations, defined as *neurosensory affection,* with an age-adjusted OR of 3.3 (95% CI; 1.6–7.0), compared to painters. No statistically significant differences were found neither in the prevalence of white fingers, nor carpal tunnel syndrome, between carpenters and painters. Among carpenters that fulfilled the criteria for *neurosensory affection*, the most common, as well as the earliest symptoms, were increased sensation of cold, numbness and pain when cold. The most common clinical findings were impaired perception of touch and increased VPT. The majority of carpenters with *neurosensory affection* reported symptoms on a daily basis, 18% reported that symptoms interfered with their daily life activities. More than 10% of young carpenters (≤ 30 y) fulfilled the criteria for *neurosensory affection,* indicating that harmful exposure still occurs*.*

### Limitations and strengths

We chose painters as a control group, as they belong to the same socioeconomic group as carpenters, and, like carpenters, have a physically demanding job with hand-intense work, often above shoulder level, but when excluding participants with possible nerve entrapment (both carpenters and painters) we did not see any major difference in the results. Another confounder regarding the control group, could be that painters had polyneuropathy from historic use of solvents. However, excluding individuals with signs of generalized neuropathy did not change the results. Furthermore during the course of the study, we discovered that some painters used vibrating tools, although to a limited extent. Furthermore, the participation rate among painters was lower than that among carpenters, and there is a risk that painters without symptoms chose not to participate. Thus, if any, these biases would result in an underestimation of the true difference in vibration injury between the groups. Thus, if any, these biases would result in an underestimation of the true difference in vibration injury between the groups. On the other hand, carpenters may be more aware of symptoms in their hands as they are well aware of their exposure to vibration, resulting in some information bias. This would cause the ORs to be somewhat overestimated.

For some participants quite some time could have passed since the occurrence of their initial symptoms for which we have to be aware of that there could be some recall bias concerning the initial symptoms.

All the participating companies were well functioning, and it is possible that their management of the working environment was somewhat better than average. To account for this, we included carpenters from other companies. The response rate among the carpenters who answered the shortened questionnaire was only about 50%. This obviously renders a high risk of selection bias concerning symptoms of HAVS. If all the carpenters who did not participate were symptom-free, the true prevalence would have been about half that reported, and about as high as that among the carpenters in the examined group. Taken together, we believe that the carpenters examined in our study were somewhat less affected by vibration than carpenters as a whole.

An important strength of this study is that the clinical examination was performed with standardised methods by trained personnel. Since the neuropathy described in HAVS is often diffuse and difficult to distinguish from other neuropathies, a test battery of different neurosensory modalities is recommended in the clinical setting. However, the test methods rely on the alertness and willingness of the tested individuals to report somatosensory stimuli [30]. Previous studies report a fair to good accordance between neurosensory symptoms and clinical findings [30]. To minimise the examination time, we only tested the perception of touch (using monofilaments) once on each tested finger, although it is recommended that this should be done three times if no response is elicited [31]. The Purdue Pegboard and aesthesiometer are recommended for obtaining ICC scores [25]. Due to practical, economic and time limitations we used 2PD and the RollTemp® instrument. However, as the same strategy was used for all the examined workers, we consider the results reliable for the purpose of this study. Both groups are manual workers, and therefore there should be no reason to suspect difference in epidermal thickness that could influence the results. Even so epidermal thickness does not seem to influence vibrotactile and thermal perception [32].

Self-reporting of *white fingers* has shown a predictive value of 80% when followed up by medical interview [33]. To allow the comparison of our results with those in other studies, we used both the ICC and the SWS criteria for staging HAVS.

### Carpenters versus painters

#### Neural manifestations

The increased prevalence of neurosensory symptoms in fingers among carpenters is well in line with the results from a previous questionnaire survey in the UK in 1997 of over 1000 men of working age [15]. Neural symptoms were found to dominate over vascular symptoms, which is also in agreement with previous studies [11, 34, 35]. Increased sensation of cold, which may be an important indication of vibration injury [36], was more common among carpenters than among painters. As many as 35% of the carpenters reported increased sensation of cold. This symptom has also been associated with medical conditions such as diabetes mellitus and rheumatic disease, but the difference remained after the exclusion of participants with these diseases. Thus, the difference is probably due to the use of vibrating tools. Cold sensitivity/cold intolerance/cold hypersensitivity is defined as abnormal aversion to cold with pain, sensory alterations, stiffness and/or colour change, but with no observable vasospasm. Pain in the fingers/hands when cold was reported among 24% of the carpenters, while the prevalence among painters (8%) was in agreement with that in the general population (5–15%) [34]. Concerning the clinical findings, we found no statistically significant differences between carpenter and painters, although the ORs were high for impaired 2PD and VPT in the carpenters. The difference between groups was greater at 250 Hz than at 125 Hz.

The ability to perceive vibrations, i.e. vibrotactile sense, is dependent on the function of cutaneous receptors and large-diameter (Aβ) afferent nerves. Some studies indicate that that larger myelinated nerves (Aβ) seem to be more vulnerable to compression than smaller myelinated nerve fibres (Aδ) and the small, unmyelinated (C) nerve fibres seem to be the most resistant to external stress [18, 37, 38]. Animal studies on vibrated rat tail have shown structural changes in blood vessels, reduced nerve fibre density and demyelisation of myelinated nerve fibres [39–41]. Nerve biopsies from vibration-exposed workers (with neural symptoms) have revealed structural changes and low myelinated nerve-fibre density [37, 42–44]. Epidemiological studies have also revealed correlations between thermal sensory impairment and cumulative exposure to vibration [45], where the perception of cold seems to be more affected than the perception of warmth [46]. In fact, the threshold for the perception of cold has been suggested as the best indicator of early neurosensory impairment since it showed greater sensitivity and specificity than the warm threshold and VPT in fingers with numbness or tingling [47]. However, in another longitudinal study on vibration-exposed workers, a low cumulative vibration dose did not significantly affect thermal perception thresholds, whereas age did [20].

In our study, impaired perception of touch and increased VPT were the two most common clinical findings among carpenters with *neurosensory affection*. In a study by Rolke et al., VPT was found to be the best method to capture neurosensory impairment in vibration-exposed workers, compared to controls [18]. The experts behind ICC suggest examination at 32 Hz and 125 Hz, but other studies have shown a greater increase in the threshold at 150 Hz than at 20 Hz among patients with HAVS [24]. In our study, VPT was more affected at 250 Hz than at 125 Hz. We therefore suggest that examination at 250 Hz be included in screening.

We found no difference in thermal perception between the carpenters and the painters, but impaired perception of cold was twice as prevalent as impaired perception of warmth among carpenters with *neurosensory affection*, when tested with the RollTempII® instrument. However, this method has not been validated, and these results should be interpreted with caution. Furthermore, the conduction velocity is much *slower* in the thin unmyelinated C fibres (which detect warmth) than in the myelinated Aδ fibres (which detect cold), which could have affected our results, leading to under-reporting of the true impairment of warmth perception.

The validity of 2PD has been questioned [48], but in the present study the prevalence of impaired 2PD was three times higher among carpenters than among painters, (although the difference was not statistically significant, due to few individuals), indicating that it may provide valuable information.

About one third of the carpenters fulfilled our criteria for *neurosensory affection*, which is of the same magnitude as reported previously for forestry workers [9]. *Neurosensory affection* was much more common among carpenters than among painters, which we attribute to the exposure of the carpenters to vibration. This difference was neither detected by the SWC nor the ICC score, although SWS stage ≥2 was more prevalent than the corresponding ICC score. These scales are based solely on the symptom of numbness, but the dominating symptom among carpenters was increased sensation of cold (67%), followed by numbness (55%), and then pain in fingers/hands when cold (45%). Therefore, we choose to set a clinical condition that we called *neurosensory affection* including impairment/sensation in Aβ, Aδ, and/or C fibres. We did not include symptoms of impaired grip strength or neuro-vegetative effects (shaking, tremors and sweating), since it has been suggested that nerve fibres other than the cutaneous afferents are involved in these conditions [26]. Only a few individuals exhibited ICC or SWS neurosensory scores indicating stage 2 or higher. Yet half of the carpenters with *neurosensory affection* reported symptoms on a daily basis, and one fifth that they interfered with daily life activities.

Both hand-intensive work and vibration exposure are risk factors for *carpal tunnel syndrome* [7, 49]. A prevalence of 7–35% has been reported among vibration-exposed workers in various epidemiological studies [11, 18, 50], with an elevated POR of 3.4 (1.4–8.3) among stoneworkers, compared to controls [51, 52]. In the clinical setting it is often difficult to distinguish neuropathy caused by compression in the carpal tunnel from more distally distributed neuropathy, as in vibration injury. Vibration perception thresholds have also been found to be increased in a group of subjects with carpal tunnel syndrome not exposed to vibration, compared to controls [53]. In our study, the prevalence of carpal tunnel syndrome was the same in both groups, and higher than expected for men in the general population [54, 55].

#### Vascular manifestations

The prevalence of white fingers among carpenters was 8%. As expected, it was higher than in among men in the general population [33]. This is in line with a nationwide study in UK, where 14% of carpenters reported white fingers [56], and with a Swedish cross sectional study, where 13% of construction workers reported white fingers [57]. In a cross sectional study on vibration-exposed stoneworkers the prevalence of white fingers was found to be 30%, compared to 4% in non-exposed controls [51]. We found no difference concerning white fingers between the carpenters and painters, which is surprising. We cannot rule out that some painters may have developed white fingers as a result of working with vibrating tools. There may also be some selection bias among the painters. To the best of our knowledge, the prevalence of white fingers among painters has not been studied previously.

Both carpenters and painters exhibited an elevated prevalence of carpal tunnel syndrome, which is risk factor for white fingers [58]. However, we found no correlation between fulfilling the criteria for carpal tunnel syndrome and reporting of white fingers.

One third of the carpenters and one tenth of the painters had noise-induced hearing loss. Other studies have shown an increased prevalence of white fingers among workers with hearing loss [59]. Although hearing loss was more common among the carpenters than the painters in the current study, we found no difference in the prevalence of white fingers between the groups.

## Conclusions

The study confirmed that carpenters reported more symptoms and exhibited a greater number of clinical findings corresponding with HAVS than painters. The OR for *neurosensory affection* was more than threefold. Also, young carpenters (≤30 years of age) showed *neurosensory affection,* which indicates that workers are currently not sufficiently protected against this kind of injury. This underlines the importance of reducing exposure to vibration and conducting regular medical check-ups to detect early signs of neural and vascular manifestations indicating HAV-injury. Special attention should be paid to the neurosensory symptoms of increased sensation of cold, pain in fingers when cold, and numbness, as these were the most common early symptoms in our study and should be addressed early in the preventive sentinel process. It is also important to test clinically for small- and large-fibre neuropathy, since the individual may be unaware of this kind of pathology.

## Data Availability

The dataset supporting the conclusions of this article is available from the corresponding author on reasonable request.

## Abbreviations

HAVS: Hand Arm Vibration Syndrome
ICC: International Consensus Criteria
SWS: Stockholm Workshop scale
VPT: Vibration Perception Threshold

## References

[1] Directive 2002/44/EC of the European Parliament and of the Council of 25 June 2002 on the minimum health and safety requirements regarding the exposure of workers to the risks arising from physical agents (vibration) (sixteenth individual Directive within the meaning of Article 16(1) of Directive 89/391/EEC) - Joint Statement by the European Parliament and the Council. 2002.

[2] AFA Insurance. Serious work-related injuries and long term sick leave 2018 (Allvarlig arbetssjukdom och långvarig sjukfrånvaro 2018; in swedish), AFA Insurance, Editor. 2018.

[3] Swedish Work Environment Authorithy (2018). The Work Environment 2017.

[4] Edlund M, Burström L, Hagberg M, Lundström R, Nilsson T, Sandén H, Wastensson G (2015). Quantitatively measured tremor in hand-arm vibration-exposed workers. Int Arch Occup Environ Health.

[5] Futatsuka M (2005). Hand arm vibration syndrome among quarry workers in Vietnam.

[6] Ando H, Noguchi R, Ishitake T (2002). Frequency dependence of hand-arm vibration on palmar sweating response. Scand J Work Environ Health.

[7] van Rijn RM, Huisstede BMA, Koes BW, Burdorf A (2009). Associations between work-related factors and the carpal tunnel syndrome--a systematic review. Scand J Work Environ Health.

[8] Wahlström J, Burström L, Hagberg M, Lundström R, Nilsson T (2008). Musculoskeletal symptoms among young male workers and associations with exposure to hand-arm vibration and ergonomic stressors. Int Arch Occup Environ Health.

[9] Bovenzi M, Rui F, Versini W, Tommasini M, Nataletti P (2004). Hand-arm vibration syndrome and upper limb disorders associated with forestry work. Med Lav.

[10] Chetter IC, Kent PJ, Kester RC (1998). The hand arm vibration syndrome: a review. Cardiovasc Surg (London, England).

[11] Nilsson T, Wahlstrom J, Burstrom L (2017). Hand-arm vibration and the risk of vascular and neurological diseases-a systematic review and meta-analysis. PLoS One.

[12] House R, Wills M, Liss G, Switzer-McIntyre S, Lander L, Jiang D (2014). The effect of hand-arm vibration syndrome on quality of life. Occup Med (Lond).

[13] Buhaug K, Moen BE, Irgens A (2014). Upper limb disability in Norwegian workers with hand-arm vibration syndrome. J Occup Med Toxicol.

[14] Budd D, Holness DL, House R (2018). Functional limitations in workers with hand-arm vibration syndrome (HAVS). Occup Med.

[15] Palmer KT, Griffin MJ, Bendall H, Pannett B, Cooper C, Coggon D. The prevalence of sensorineural symptoms attributable to hand-transmitted vibration in Great Britain: a national postal survey. Am J Ind Med. 2000;38(1):99–107. 10.1002/1097-0274(200007)38:1<99::AID-AJIM11>3.0.CO;2-X. 10.1002/1097-0274(200007)38:1<99::aid-ajim11>3.0.co;2-x 10861771

[16] Gemne G (1997). Diagnostics of hand-arm system disorders in workers who use vibrating tools. Occup Environ Med.

[17] Mahbub MH (2015). A systematic review of diagnostic performance of quantitative tests to assess musculoskeletal disorders in hand-arm vibration syndrome. Ind Health.

[18] Rolke R, Rolke S, Vogt T, Birklein F, Geber C, Treede RD, Letzel S, Voelter-Mahlknecht S (2013). Hand-arm vibration syndrome: clinical characteristics, conventional electrophysiology and quantitative sensory testing. Clin Neurophysiol.

[19] Lindsell CJ (2003). Griffin MJ, normative data for vascular and neurological tests of the hand -arm vibration syndrome. Int Arch Occup Environ Health.

[20] Lundstrom R (2018). Long-term effect of hand-arm vibration on thermotactile perception thresholds. J Occup Med Toxicol.

[21] FHV Metodik, Vibrationer. 2019: p. http://fhvmetodik.se/wp-content/uploads/2014/03/Instruktion-MKA-vibration-Skanör20191024.pdf.

[22] Lindsell CJ, Griffin MJ (1999). Thermal thresholds, vibrotactile thresholds and finger systolic blood pressures in dockyard workers exposed to hand-transmitted vibration. Int Arch Occup Environ Health.

[23] Rolke R, Baron R, Maier C, Tölle TR, Treede DR, Beyer A, Binder A, Birbaumer N, Birklein F, Bötefür IC, Braune S, Flor H, Huge V, Klug R, Landwehrmeyer GB, Magerl W, Maihöfner C, Rolko C, Schaub C, Scherens A, Sprenger T, Valet M, Wasserka B (2006). Quantitative sensory testing in the German research network on neuropathic pain (DFNS): standardized protocol and reference values. Pain.

[24] Lundborg G, et al. Vibrotactile function of the hand in compression and vibration-induced neuropathy. Sensibility index--a new measure. Scand J Plast Reconstr Surg Hand Surg. 1992;26(3). 10.3109/02844319209015271 1335164

[25] Poole CJM, Bovenzi M, Nilsson T, Lawson IJ, House R, Thompson A, Youakim S (2019). International consensus criteria for diagnosing and staging hand-arm vibration syndrome. Int Arch Occup Environ Health.

[26] Brammer AJ, Taylor W, Lundborg G (1987). Sensorineural stages of the hand-arm vibration syndrome. Scand J Work Environ Health.

[27] Griffin MJ, Bovenzi M (2002). The diagnosis of disorders caused by hand-transmitted vibration: Southampton workshop 2000. Int Arch Occup Environ Health.

[28] Nordander C, Ohlsson K, Åkesson I, Arvidsson I, Balogh I, Hansson GÅ, Strömberg U, Rittner R, Skerfving S (2009). Risk of musculoskeletal disorders among females and males in repetitive/constrained work. Ergonomics.

[29] Ohlsson K (1994). An assessment of neck and upper extremity disorders by questionnaire and clinical examination. Ergonomics.

[30] Lundström R, Nilsson T, Hagberg M, Burström L (2008). Grading of sensorineural disturbances according to a modified Stockholm workshop scale using self-reports and QST. Int Arch Occup Environ Health.

[31] National Assessement Manual for assessement for hand function after nerve repair, Version 1, 2018. 2018: p. HAKIR Handkirurgiskt Kvalitetsregister.

[32] Lundstrom R (2018). Vibrotactile and thermal perception and its relation to finger skin thickness. Clin Neurophysiol Pract.

[33] Leppert J, Aberg H, Ringqvist I, Sörensson S (1987). Raynaud's phenomenon in a female population: prevalence and association with other conditions. Angiology.

[34] Stjernbrandt, A., Pettersson, H., Liljelind, I. et al. , Raynaud’s phenomenon in Northern Sweden: a population-based nested case–control study. Rheumatol Int (2019) 39: 265andt, A., Pettersson, H., Liljelind, I. et al. Rheumatol Int (2019) 39: 265, 2019. 10.1007/s00296-018-4133-y 30128730

[35] Edlund M, Burström L, Gerhardsson L, Lundström R, Nilsson T, Sandén H, Hagberg M (2014). A prospective cohort study investigating an exposure-response relationship among vibration-exposed male workers with numbness of the hands. Scand J Work Environ Health.

[36] Carlsson D, Wahlström J, Burström L, Hagberg M, Lundström R, Pettersson H, Nilsson T (2018). Can sensation of cold hands predict Raynaud’s phenomenon or paraesthesia?. Occup Med.

[37] Dahlin LB, Sandén H, Dahlin E, Zimmerman M, Thomsen N, Björkman A (2014). Low myelinated nerve-fibre density may lead to symptoms associated with nerve entrapment in vibration-induced neuropathy. J Occup Med Toxicol.

[38] Dahlin LB (1989). Effects of nerve compression or ischaemia on conduction properties of myelinated and non-myelinated nerve fibres. An experimental study in the rabbit common peroneal nerve. Acta Physiol Scand.

[39] Goenka S (2013). Dependence of vascular damage on higher frequency components in the rat-tail model. Ind Health.

[40] Lundborg G, Dahlin LB, Hansson HA, Kanje M, Necking LE (1990). Vibration exposure and peripheral nerve fiber damage. J Hand Surg [Am].

[41] Loffredo MA, Yan JG, Kao D, Zhang LL, Matloub HS, Riley DA (2009). Persistent reduction of conduction velocity and myelinated axon damage in vibrated rat tail nerves. Muscle Nerve.

[42] Stromberg T, Dahlin LB, Brun A, Lundborg G (1997). Structural nerve changes at wrist level in workers exposed to vibration. Occup Environ Med.

[43] Takeuchi T (1986). Pathological changes observed in the finger biopsy of patients with vibration-induced white finger. Scand J Work Environ Health.

[44] Takeuchi T, Takeya M, Imanishi H (1988). Ultrastructural changes in peripheral nerves of the fingers of three vibration-exposed persons with Raynaud's phenomenon. Scand J Work Environ Health.

[45] Nilsson T, Lundström R (2001). Quantitative thermal perception thresholds relative to exposure to vibration. Occup Environ Med.

[46] Hirosawa I, Nishiyama K, Watanabe S (1992). Temporary threshold shift of temperature sensation caused by vibration exposure. Int Arch Occup Environ Health.

[47] Ye Y, Griffin MJ (2018). Assessment of thermotactile and vibrotactile thresholds for detecting sensorineural components of the hand-arm vibration syndrome (HAVS). Int Arch Occup Environ Health.

[48] Lundborg G, Rosen B (2004). The two-point discrimination test--time for a re-appraisal?. J Hand Surg (Br).

[49] Palmer KT, Harris EC, Coggon D (2007). Carpal tunnel syndrome and its relation to occupation: a systematic literature review. Occup Med (Lond).

[50] Barcenilla A, March LM, Chen JS, Sambrook PN (2012). Carpal tunnel syndrome and its relationship to occupation: a meta-analysis. Rheumatology (Oxford).

[51] Bovenzi M (1994). Hand-arm vibration syndrome and dose-response relation for vibration induced white finger among quarry drillers and stonecarvers. Italian study group on physical hazards in the stone industry. Occup Environ Med.

[52] Harris-Adamson C, Eisen EA, Neophytou A, Kapellusch J, Garg A, Hegmann KT, Thiese MS, Dale AM, Evanoff B, Bao S, Silverstein B, Gerr F, Burt S, Rempel D (2016). Biomechanical and psychosocial exposures are independent risk factors for carpal tunnel syndrome: assessment of confounding using causal diagrams. Occup Environ Med.

[53] Flondell M, Rosén B, Andersson G, Schyman T, Dahlin LB, Björkman A (2017). Vibration thresholds in carpal tunnel syndrome assessed by multiple frequency vibrometry: a case-control study. J Occup Med Toxicol.

[54] Atroshi I, Englund M, Turkiewicz A, Tägil M, Petersson IF (2011). Incidence of physician-diagnosed carpal tunnel syndrome in the general population. Arch Intern Med.

[55] Atroshi I, Gummesson C, Johnsson R, Ornstein E, Ranstam J, Rosén I (1999). Prevalence of carpal tunnel syndrome in a general population. JAMA.

[56] Palmer KT, Griffin MJ, Syddall H, Pannett B, Cooper C, Coggon D (2001). Risk of hand-arm vibration syndrome according to occupation and sources of exposure to hand-transmitted vibration: a national survey. Am J Ind Med.

[57] Burstrom L (2010). White fingers, cold environment, and vibration--exposure among Swedish construction workers. Scand J Work Environ Health.

[58] Hartmann P, Mohokum M, Schlattmann P (2012). The association of Raynaud's syndrome with carpal tunnel syndrome: a meta-analysis. Rheumatol Int.

[59] Pettersson H, Hagberg M, Nilsson T, Burström L, Lundström R (2012). Noise and hand-arm vibration exposure in relation to the risk of hearing loss. Noise Health.

